# Supplementary material for: Maintenance of niche architecture requires actomyosin and enables proper stem cell signaling and oriented division in the Drosophila testis
Source: Development. 2025 Jan 9;152(1):dev204498. doi: 10.1242/dev.204498 (PMC11795290; doi:10.1242/dev.204498)
Supplement: Supplementary information [file develop-152-204498-s1.pdf]

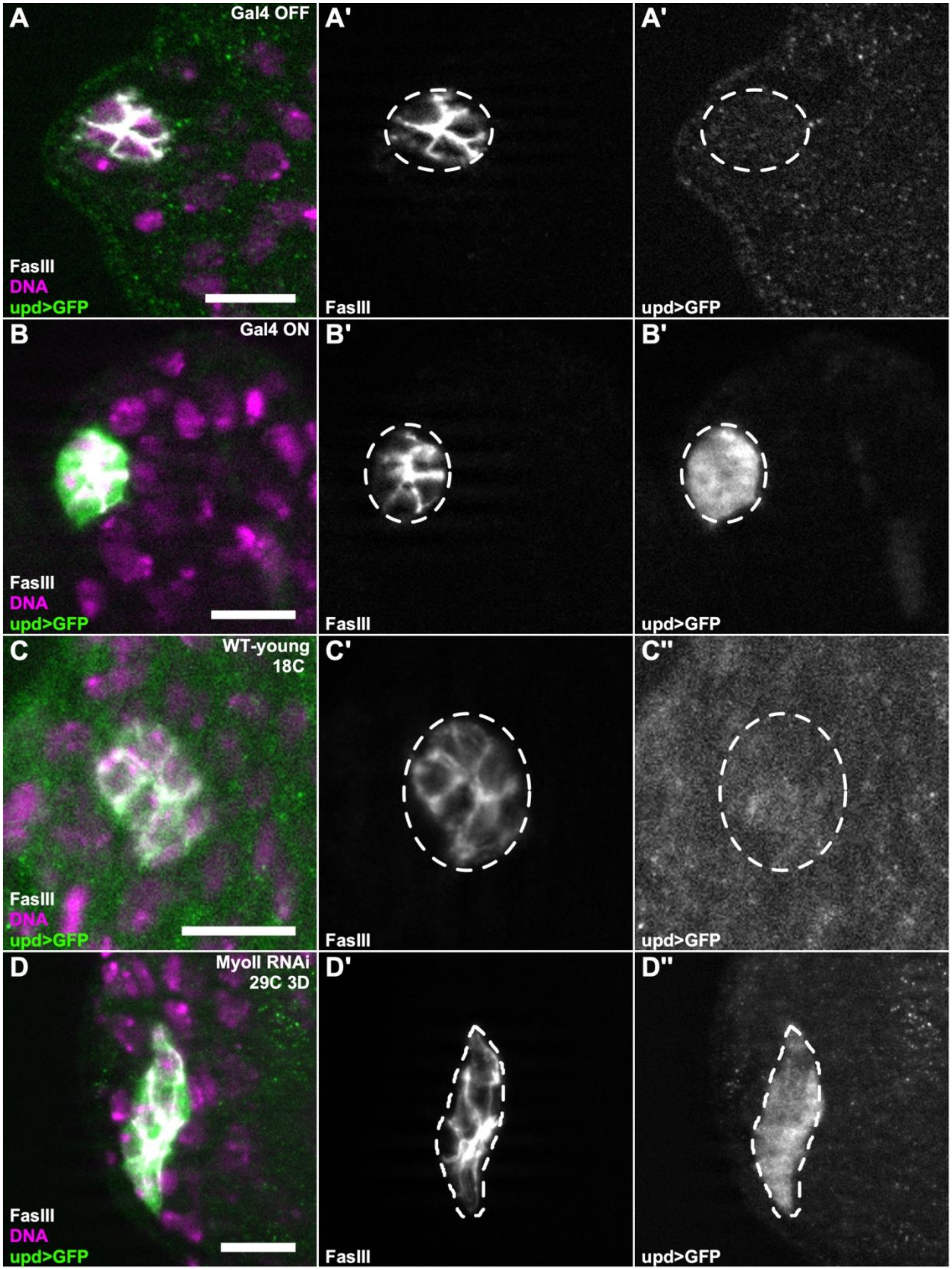

Development • Supplementary information

Fig. S1. Gal4 inhibited by Gal80 at 18 degrees

(A-B) Immunostained young testis kept at 18 degrees until dissection (A) or upshifted to 29 during late pupation and dissected after 3 days at 29 (B); (A,B) DNA (magenta), FasIII (white), upd>GFP (green); (A',B') FasIII only; (A'',B'') upd>GFP only, no GFP expression in the niche because the Gal4 is off at 18 degrees (A'') compared to GFP expression nicesly restricted to niche cells after Gal4 induction (B'').

(C-D) Immunostained WT young testis with MyoII RNAi in the background, kept at 18 degrees until dissection (C) or upshifted to 20 during late pupation and dissected after 3 days (D); (C,D) DNA (magenta), FasIII (white), upd>GFP (green); (C',D') FasIII only; (C'',D'') upd>GFP only, no GFP expression and no niche defects with Gal4 inhibited (C'') compared to GFP expression is nicely restricted to niche cells after Gal4 induction coupled with structural defects caused by Gal4 induction at 29 degrees.

Development • Supplementary information

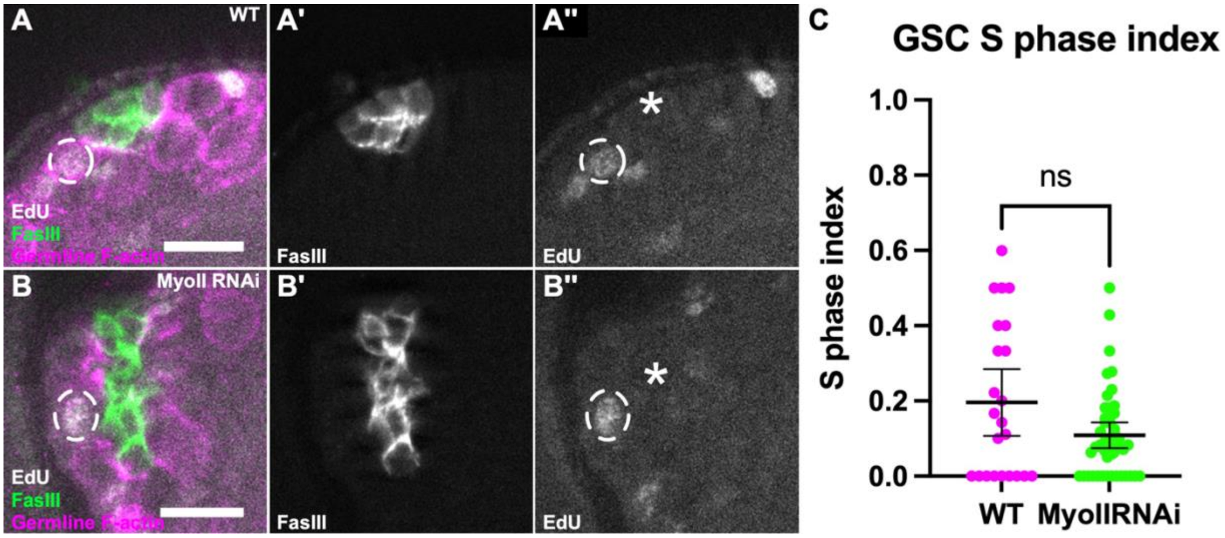

Fig. S2. Depletion of MyoII does not induce S phase entry niche cells and does not change S phase frequency in GSCs

(A-B) Immunostained WT (A) and MyoII RNAi (B) testis; (A,B) nanos-lifeactdTom, FasIII (green), EdU(white); (A’,B’) FasIII only; (A’’,B’’) EdU only; niche represented by white asterisk, EdU positive GSC indicated by white dotted circle.

(C) Quantification of S phase index of GSCs shows no difference in S phase entry between GSCs in a WT background compared to GSCs in a MyoII knockdown background (ns).

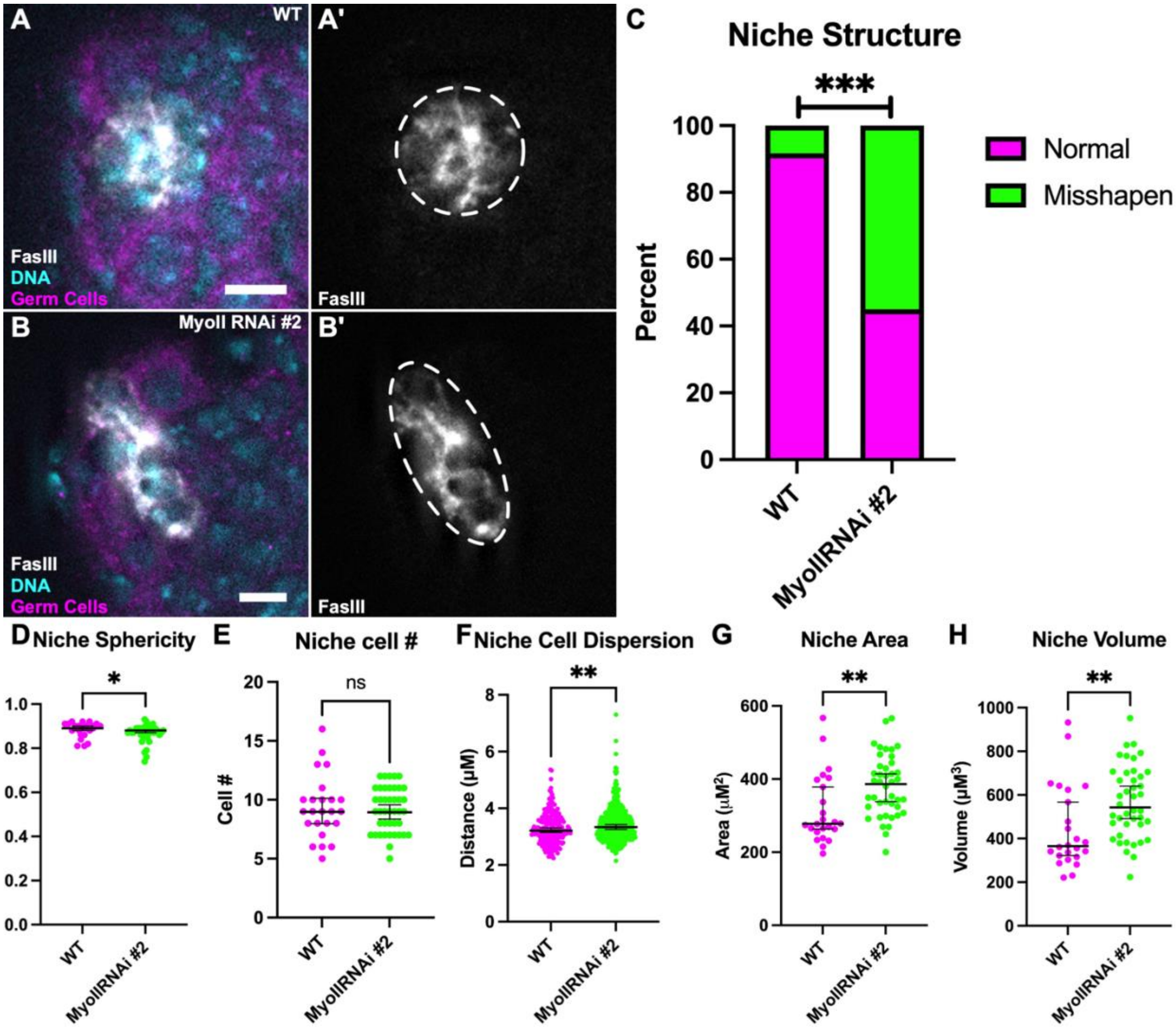

Fig. S3. MyoII RNAi #2 has niche shape defects

(A-B) Immunostained WT (A) and MyoIIRNAi (B) testis; (A, B) vasa (magenta), FasIII (white), Hoescht (cyan); (A',B') FasIII only; niche shape represented by white dotted line.

(C) Quantification of niche disassembly shows MyoII RNAi niches are severely misshapen compared to WT niches (WT: 2/24 niches misshapen, MyoIIRNAi#2: 22/40 niches misshapen, ***p=0.0002, Fisher's exact test).

(D) Quantification of niche sphericity between WT and MyoIIRNAi niches (*p=0.0316, Mann- Whitney test).

(E) Quantification of niche cell number show no difference between WT and MyoIIRNAi niches (ns).

(F) Quantification of the average distance of a niche cell and its 3 nearest neighbors show increased distance between MyoIIRNAi niche cells compared to WT (**p=0.0063, Mann- Whitney test).

(G-H) Quantification of the niche area (G) and volume (H) shows an increase in niche area in MyoIIRNAi niches compared to WT (**p=0.0012(G), **p=0.0022(H), Mann-Whitney test).

Development • Supplementary information

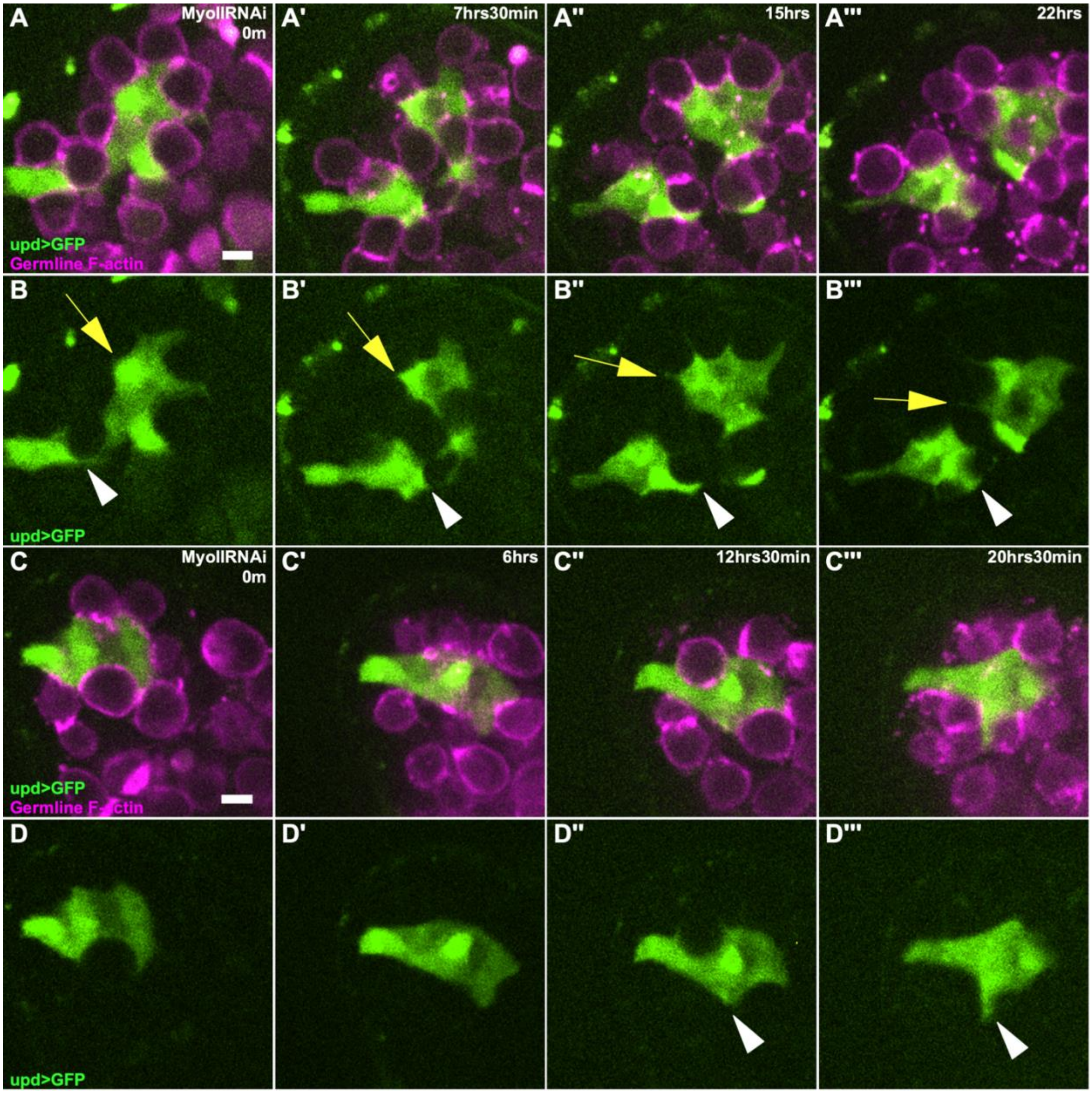

Fig. S4. Niches lose compactness and extend protrusions upon depletion of MyoII (A-D) Time-lapse of niche disassembly of MyoIIRNAi niche; (A-A''', C-C''') upd>GFP (green), nanos-lifeacttdTom (magenta); (B-B''', D-D''') upd>GFP only, dotted line represents niche shape at each time point. Niche cells extend protrusions that extend over time (B-B''', white arrowheads and yellow arrows, D-D''' white arrowheads).

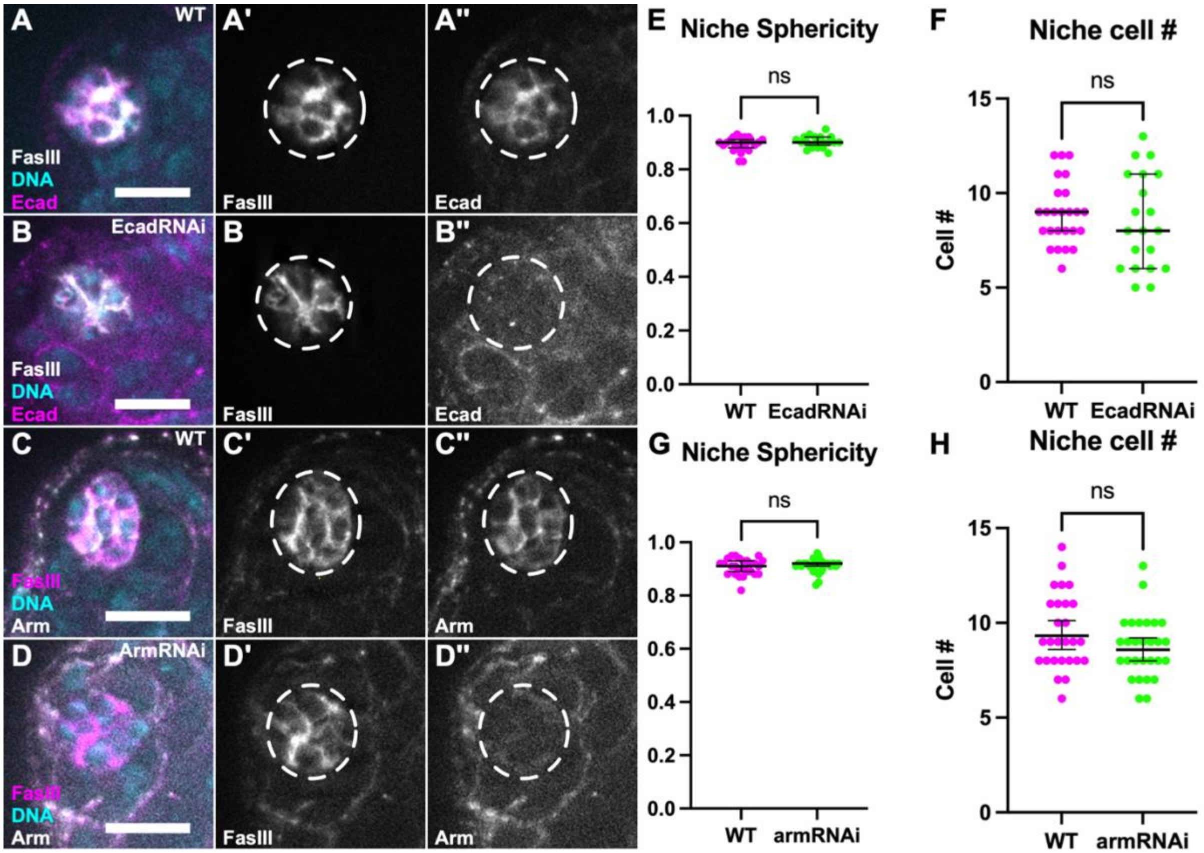

Fig. S5. Depletion of E-cadherin and Beta-catenin do not affect niche shape

(A-B) Immunostained WT (A) and E-cadherin RNAi (B) testis; (A,B) E-cadherin (magenta), FasIII (white), Hoescht (cyan); (A’,B’) FasIII only; (A’’,B’’) E-cadherin only; niche shape represented by white dotted line, E-cadherin is clearly lost from niche, however the niche is still compact in structure (B’’).

(C-D) Immunostained WT (C) and Arm/Beta-catenin RNAi (D) testis; (C) FasIII (magenta), Arm/Beta-catenin (white), Hoescht (cyan); (C’,D’) FasIII only; (C’’,D’’) Arm/Beta-catenin only; niche shape represented by white dotted line; arm/Beta-catenin is clearly lost from the niche, however the niche is still compact in structure.

(E,G) Quantification of sphericity between age-matched control and E-cadherin RNAi (E) or arm/Beta-catenin RNAi niches (G) (ns).

(F,H) Quantification of niche cell number show no difference between age-matched control and E-cadherin RNAi (F) or arm/Beta-catenin (H) niches (ns).

Development • Supplementary information

Table S1. Key Reagent Table

Reagent or Resource	Source	Identifier
Genetic Reagent (D. melanogaster)		
w1118	Bloomington Drosophila Stock Center	BL#3605; RRID:BDSC_3605
sqh::GFP3x	Pinheiro et al 2017	Bellaiche
UAS-GFP::Zipper	D. Kiehart	Curr. Biol. 15, 2208–2221
upd-Gal4	Bloomington Drosophila Stock Center	BDSC_26796
upd-Gal4;tubGal80ts	recombinant made from updGal4 (E.Matunis) and tubGal80ts	
nos5'- Lifeact-tdtomato p2a tdkatushka2 Caax nos3'	Lin et al. 2020	N/A
zip::GFP, nosred	Vienna Drosophila Resource Center, Lin et al. 2020	VRDC#115082
Ubi::Rok-GFP	Zallen Lab	N/A
UAS-zipper-RNAi	Bloomington Drosophila Stock Center	BL#65947
UAS-zipper-RNAi	Bloomington Drosophila Stock Center	BL#37480
UAS-ROK-RNAi	Bloomington Drosophila Stock Center	BL#34324
UAS-shg-RNAi	Bloomington Drosophila Stock Center	BL#32904
UAS-arm-RNAi	Vienna Drosophila Resource Center	VDRC# 107344
Antibodies		
Rabbit polyclonal anti Vasa	R. Lehmann	N/A
Mouse monoclonal anti Fasciclin III	Developmental Studies Hybridoma Bank	DSHB:7G10; RRID:AB_528238
Rabbit polyclonal anti STAT92E	E. Bach	N/A
Rabbit polyclonal anti RFP	Abcam	ab62341; RRID:AB_945213
Rat monoclonal anti DE-cadherin	Developmental Studies Hybridoma Bank	DSHB:DCAD2 RRID: AB_528120
Rat monoclonal anti DN-cadherin	Developmental Studies Hybridoma Bank	DSHB:DN-ex #8:AB_528121
Chick polyclonal anti GFP	Aves Labs	Cat#GFP-1020; RRID: AB_2307313
Rabbit PH3	Upstate Biotech	lot 15420, cat #06-570

Mouse gamma tubulin	Sigma	GTU-88, T6557; RRID: AB_477584
Myosin II (zipper/HC)	D. Kiehart	N/A
B-catenin (armadillo)	Developmental Studies Hybridoma Bank	DSHB: N2 7A1; RRID: AB_528089
Normal Donkey Serum (NDS)	Jackson Immunoresearch Laboratories	Cat#: 017-000-121; RRID: AB_2337258
Alexafluor Secondary Antibodies (488, 647)	Molecular Probes	
Cy3 Affinipure Secondary Antibodies	Jackson ImmunoResearch	Cat#: 711-165-152; RRID: AB_2307443
Chemical Compound		
Poly Lysine	Sigma	Cat#P-1274; CAS Number 25988-63-0; PubChem Substance ID 24898180
FBS	GIBCO	Cat#10082
penicillin/streptomycin	Corning	Cat#30-002-Cl
Schneider's Insect Media	GIBCO	Cat#21720-024
Insulin,Bovine	Sigma	Cat#I0516
Para-Formaldehyde (PFA), 16%	Electron Microscopy Sciences	Cat#15710
PFA, 40%	Electron Microscopy Sciences	Cat#15715-S
Hoechst	Sigma	Cat#33342; CAS Number: 875756-97-1
Propyl-gallate	Sigma Aldrich	SKU: P3130; CAS Number 121-79-9; PubChem Substance ID 24898394
Normal Donkey Serum (NDS)	Jackson Immunoresearch Laboratories	017-000-121; RRID: AB_2337258
Ringer's solution	Other	doi:10.1101/pdb.rec12409
DMSO	Molecular Probes	Cat#D12345
Colcemid	Millipore Sigma	CAS-No: 477-30-5
Commercial Assays		
Click-iT EdU	ThermoFisher	Cat#: C10419
Software		
FIJI (Image J)	www.fiji.sc	N/A
Image J	www.imagej.nih.gov/ij/	N/A
EZ Fig	Image J Plugin	
Axio-Vision Imaging Software	Zeiss	v4.8.1
Graphpad Prism	Graphpad Software	v7.0; RRID:SCR_002798

Development • Supplementary information

Imaris	https://imaris.oxinst.com/products/imaris-for-core-facilities	
Other		
Matek imaging dish	Thermofisher	P35G-1.5-14-C
AxioCamHR	Zeiss	
40x/ 1.2 NA water immersion objective	Zeiss	
60X / 1.4 NA SI Oil immersion objective	Olympus/ Evident	Lenhart Lab
Olympus IX83 Yokagama CSU-10 spinning disk scan head	Olympus/ Evident	Lenhart Lab
Hamamatsu EM-CCD camera	Olympus/ Evident	Lenhart Lab

Development • Supplementary information
