# Supplementary material for: Maintenance of niche architecture requires actomyosin and enables proper stem cell signaling and oriented division in the Drosophila testis
Source: Development. 2025 Jan 9;152(1):dev204498. doi: 10.1242/dev.204498 (PMC11795290; doi:10.1242/dev.204498)
Supplement: Supplementary file 1 [file develop-152-204498_review_history.pdf]

Maintenance of niche architecture requires actomyosin and enables proper stem cell signaling and oriented division in the Drosophila testis

Gabriela S. Vida, Elizabeth Botto and Stephen DiNardo
DOI: 10.1242/dev.204498

Editor: Thomas Lecuit

Review timeline
Original submission: 4 June 2024
Editorial decision: 28 June 2024
Resubmission: 6 November 2024
Accept: 20 November 2024

Original submission

First decision letter

MS ID#: DEVELOP/2024/203125

MS TITLE: Niche cytoskeletal architecture is required for proper stem cell signaling and oriented division in the Drosophila testis
AUTHORS: Gabriela S Vida, Elizabeth Botto, and Stephen DiNardo

Dear Steve,

I have now received all the referees reports on the above manuscript, and have reached a decision. The referees' comments are appended below, or you can access them online: please go to BenchPress and click on the 'Manuscripts with Decisions' queue in the Author Area.

The overall evaluation is very positive and we would like to publish a revised manuscript in Development, provided that the referees' minor comments can be satisfactorily addressed. Please attend to all of the reviewers' comments in your revised manuscript and detail them in your point-by-point response. If you do not agree with any of their criticisms or suggestions explain clearly why this is so. If it would be helpful, you are welcome to contact us to discuss your revision in greater detail. Please send us a point-by-point response indicating your plans for addressing the referees' comments, and we will look over this and provide further guidance.

Reviews

Reviewer 1 Advance Summary and Potential Significance to Field:

The Drosophila testis is a well-established system for studying stem cells in vivo. Here Vida et al. use this tissue to study the role of cytoskeletal architecture on niche function. This group recently showed that actomyosin contractility is important for formation of this niche during development. Here, Vida et al. examine roles for actomyosin contractility during the steady state operation of the adult niche.

They begin by showing that reporters for two key regulators of actomyosin contractility, Myosin II and Rho kinase, are enriched at the interface between niche cells and stem cells

© 2024. Published by The Company of Biologists under the terms of the Creative Commons Attribution License (https://creativecommons.org/licenses/by/4.0/).

(as opposed to the interfaces where niche cells contact one another). Next, a requirement for both factors in maintaining the correct morphology and function of this niche is shown via conditional RNAi-mediated knockdown. The carefully quantified data show that depletion of Myosin II or Rho kinase does not alter the number of niche cells or their identity, but does decrease the sphericity of the niche while increasing niche area, volume and dispersion. Interestingly some niches even split apart, and niche phenotypes progress with time. The authors also make excellent use of time lapse imaging of cultured testes to reveal that niches lacking myosinII undergo shape changes over a 24 hour period. The newly dynamic behavior of individual mutant niche cells strongly supports the idea that altered cytoskeletal contractility drives the phenotypes described above. Downstream consequences to surrounding germline stem cells after myosin II knockdown are also carefully quantified. As expected, given the larger niche area, stem cell number increases. Unexpectedly the levels of the stem cell factor STAT decrease compared to nearby germ cells that do not contact the niche. These germline stem cells undergo more frequent mis-oriented divisions and symmetric renewals as assayed by live imaging, revealing important changes to their behavior.

Taken together, these data strongly support the central hypothesis that the cytoskeletal architecture of the niche cells themselves non-autonomously regulates stem cell signaling and divisions. Each figure is very nicely presented, with careful attention to both clarity throughout. Many of the experiments are technically challenging, yet carefully executed with attention to appropriate statistical analysis. Since little is known about how the organization of niche shape, structure or organization is regulated in general, Vida et al's clear demonstration that niche structure impacts stem cell dynamics during the steady state function of this well- characterized model stem cell niche is impactful and interesting. Researchers using other systems will likely apply this forward-thinking concept in additional contexts.

The data are well-controlled and strongly support the conclusions. No additional suggestions. The carefully laid out "limitations" paragraph was also appreciated.

Reviewer 1 Comments for the Author:

The data are well-controlled and strongly support the conclusions. No additional suggestions. The carefully laid out "limitations" paragraph was also appreciated.

Reviewer 2 Advance Summary and Potential Significance to Field:

This study by Vida et al shows that a stem cell niche in the Drosophila testes autonomously requires actomyosin activity to maintain its shape, to optimize the number of affiliated germline stem cells, and to direct properly oriented asymmetric germline stem cell (GSC) divisions. Surprisingly, the authors show that activated myosin is required in the niche for the centrosome orientation checkpoint in the GSCs. This is a very interesting set of findings since not much is known about molecular drivers of niche architecture or how the niche structure impacts stem-ness, and these mechanisms may be conserved in other contexts. Thus, the paper makes an important and novel contribution to this topic.

Reviewer 2 Comments for the Author:

Overall, the experiments have been carefully performed, the data are convincing, and the work is presented clearly in general. I only have minor suggestions for improvement.

Minor suggestions:
- In the title, I don't think the phrase "cytoskeletal architecture" properly reflects what the study shows because it implies characterization of the structure/organization of the cytoskeleton instead of the niche itself. The authors might consider something like: "Maintenance of niche architecture requires actomyosin and enables proper stem cell signaling and oriented division…"

© 2024. Published by The Company of Biologists under the terms of the Creative Commons Attribution License (https://creativecommons.org/licenses/by/4.0/).

- The abstract implies that actomyosin contractility (AMC) is important, but the data for this are a bit indirect and they do not make strong claims about it in the results. It is likely true that AMC is important since both myosin and its activator Rok are required, but to strengthen this claim and keep it in the abstract, the authors should provide more data. They could examine the dynamics of sqh-GFP by live imaging since there can be flashes that are thought to represent activation, or stain the niche for phospho-Myosin localization in the control and Rok knockdown, or express a phosphomimetic sqh mutant in the niche and assay the phenotype.

- The polarity of cadherins in the niche or niche-GSC interface is a bit hard to see since the niche is dramatically misshapen in the examples (Fig 5B and D). Also, were these a 10-day disruption or shorter time? I was unclear how this was quantified. The authors say they measured across 3 cell interfaces (niche/niche interface or niche/GSC interface). But how did they account for the differences in size of the interfaces? It seems like niche/GSC interfaces would always be longer and thus return a higher value of total intensity.

- In figure 6, the authors show a disruption to STAT signaling when myosin is disrupted, which is an important read out for stem-cell-character. The difference is significant but small, so the details of how this experiment was conducted may be important. Specifically, since upd-Gal4 is an insertion in/near upd (which could lead to disruption) and the controls do not have the Gal 4 (if I understood the methods correctly) – is it possible some of the reduced STAT signaling is due to upd disruption or a genetic interaction with upd? An additional control or two would help clear up this possibility: for example, a different Gal4 driver, or upd-Gal4 alone, or no temperature upshift with the same genotype.

- Line 388 – wording is confusing – not sure what they mean by “lower frequency” (eg, what compared to what?)

- For the case shown in Figure 8 where there is a “double oriented” centrosome, there are niche cells on both sides, which means it may be positioned correctly (twice). Does this “double oriented” case always have two niche cells or wrapping around of the niche?

- I understand the authors were trying to minimize detail to simplify the presentation, but think it would be helpful to have a bit more detail in the figures or figure legends, including the Gal4 driver and the timing of the temperature shifts.

-Figure 1 legend - not sure what the authors mean by “displayed in 3D” -is this a maximum projection?

-Figure 2 legend, line 871 – should say "distance of a niche cell nucleus and three nearest-neighbor nuclei".

- The data on MyoII localization in Figure 3 J and K is hard to see with the overlaid dotted lines, especially for MyoII in J’’ and K’’ – perhaps these lines can be moved to the first panel.

- Figure 7 – what is the difference between A and D? Are both needed?

- The niche looks pretty different in Supplemental Figure 4 compared to Figure 7- the cells look much more stellate and protrusive– why? Is it just because this image is less magnified or is the phenotype this variable?

Author response to reviewers' comments

***** Reviewer 2 Advance Summary and Potential Significance to Field:
This study by Vida et al shows that a stem cell niche in the Drosophila testes autonomously requires actomyosin activity to maintain its shape, to optimize the number of affiliated germline stem cells, and to direct properly oriented asymmetric germline stem cell (GSC) divisions. Surprisingly, the authors show that activated myosin is required in the niche for the centrosome orientation

© 2024. Published by The Company of Biologists under the terms of the Creative Commons Attribution License (https://creativecommons.org/licenses/by/4.0/).

checkpoint in the GSCs. This is a very interesting set of findings since not much is known about molecular drivers of niche architecture or how the niche structure impacts stem-ness, and these mechanisms may be conserved in other contexts. Thus, the paper makes an important and novel contribution to this topic.

Reviewer 2 Comments for the Author:
Overall, the experiments have been carefully performed, the data are convincing, and the work is presented clearly in general. I only have minor suggestions for improvement.

Minor suggestions: - In the title, I don't think the phrase "cytoskeletal architecture" properly reflects what the study shows because it implies characterization of the structure/organization of the cytoskeleton instead of the niche itself. The authors might consider something like: "Maintenance of niche architecture requires actomyosin and enables proper stem cell signaling and oriented division..."

- We took the referee's suggestions and changed the title to what was suggested.

- The abstract implies that actomyosin contractility (AMC) is important, but the data for this are a bit indirect and they do not make strong claims about it in the results. It is likely true that AMC is important since both myosin and its activator Rok are required, but to strengthen this claim and keep it in the abstract, the authors should provide more data. They could examine the dynamics of sqh-GFP by live imaging since there can be flashes that are thought to represent activation, or stain the niche for phospho-Myosin localization in the control and Rok knockdown, or express a phosphomimetic sqh mutant in the niche and assay the phenotype.

- We took the referee's suggestion and examined sqh-GFP captured live at short intervals. This indeed revealed dynamic puncta along niche-GSC interfaces consistent with episodes of active contractility. This data is included in new Figure 2 A and B panels.

- The polarity of cadherins in the niche or niche-GSC interface is a bit hard to see since the niche is dramatically misshapen in the examples (Fig 5B and D). Also, were these a 10-day disruption or shorter time? I was unclear how this was quantified. The authors say they measured across 3 cell interfaces (niche/niche interface or niche/GSC interface). But how did they account for the differences in size of the interfaces? It seems like niche/GSC interfaces would always be longer and thus return a higher value of total intensity.

- The values we report are not total intensity, but rather are normalized for junction lengths. This is now clarified in the methods section and figure legend to be more explicit.

- In figure 6, the authors show a disruption to STAT signaling when myosin is disrupted, which is an important read out for stem-cell-character. The difference is significant but small, so the details of how this experiment was conducted may be important. Specifically, since upd-Gal4 is an insertion in/near upd (which could lead to disruption) and the controls do not have the Gal 4 (if I understood the methods correctly) – is it possible some of the reduced STAT signaling is due to upd disruption or a genetic interaction with upd? An additional control or two would help clear up this possibility: for example, a different Gal4 driver, or upd-Gal4 alone, or no temperature upshift with the same genotype.

- We took the referee's suggestion, repeating this experiment using no temperature upshifts with the same genotype as controls. The data are essentially the same, with STAT signaling disrupted and more germ cells responding to signaling. This data replaces previous analysis in Figure 6.

- Line 388 – wording is confusing – not sure what they mean by "lower frequency" (eg, what compared to what?)

- We clarified this in the text. We tried to articulate that there were not only fewer ROK RNAi niches that were defective, but that this phenotype was also less severe compared to MyoII RNAis.

© 2024. Published by The Company of Biologists under the terms of the Creative Commons Attribution License (https://creativecommons.org/licenses/by/4.0/).

- For the case shown in Figure 8 where there is a "double oriented" centrosome, there are niche cells on both sides, which means it may be positioned correctly (twice). Does this "double oriented" case always have two niche cells or wrapping around of the niche?

- We rewrote this section to state that each of the two centrosomes may indeed be captured in the GSC cortex at an interface with a niche. We point out that division in such instances will yield two cells that may have stem cell character, rather than the usual one stem cell and one differentiating daughter cell.

- I understand the authors were trying to minimize detail to simplify the presentation, but think it would be helpful to have a bit more detail in the figures or figure legends, including the Gal4 driver and the timing of the temperature shifts.

- As requested, we added the Gal4 driver and the length of knockdown to the text in each of the figure legends.

-Figure 1 legend - not sure what the authors mean by "displayed in 3D" -is this a maximum projection?

- We displayed the testis niches in a 3D software and took one snapshot of the niche with its surface displayed in an inset.

-Figure 2 legend, line 871 – should say "distance of a niche cell nucleus and three nearest-neighbor nuclei".

- We clarified the writing in this line, along with the writing in the other figure legends where applicable.

- The data on MyoII localization in Figure 3 J and K is hard to see with the overlaid dotted lines, especially for MyoII in J'' and K'' – perhaps these lines can be moved to the first panel.

- As requested, we removed the line.

- Figure 7 – what is the difference between A and D? Are both needed?

- The panels in figure 7 A and D were both controls. We therefore removed panel D and readjusted the figure paneling.

- The niche looks pretty different in Supplemental Figure 4 compared to Figure 7- the cells look much more stellate and protrusive– why? Is it just because this image is less magnified or is the phenotype this variable?

- The phenotype is variable to some degree. There are instances where some niches show more protrusive qualities than others, often times when they are more dispersed.

Resubmission

First decision letter

MS ID#: DEVELOP/2024/204498

MS Title: Maintenance of niche architecture requires actomyosin and enables proper stem cell signaling and oriented division in the Drosophila testis

Authors: Gabriela S Vida; Elizabeth Botto; Stephen DiNardo
Article Type: Research Article

© 2024. Published by The Company of Biologists under the terms of the Creative Commons Attribution License (https://creativecommons.org/licenses/by/4.0/).

I looked carefully at your revised manuscript and in light of the additional experiments and responses to the requests of clarifications I am happy to tell you that your manuscript has been accepted for publication in Development, pending our standard publication integrity checks.

© 2024. Published by The Company of Biologists under the terms of the Creative Commons Attribution License (https://creativecommons.org/licenses/by/4.0/).
